# Changing Levels of Dental Caries over 30 Years among Children in a Country of Central and Eastern Europe – The Case of Hungary

**DOI:** 10.3290/j.ohpd.a44322

**Published:** 2020-02-12

**Authors:** Judit Szöke, Poul Erik Petersen

**Affiliations:** a Professor, Semmelweis University of Medicine, Faculty of Dentistry, Budapest, Hungary. Study design, data collection, analysis, wrote manuscript.; b Professor, World Health Organization Collaborating Centre for Community Oral Health Programmes and Research, University of Copenhagen, Denmark. Study design, data collection, analysis, wrote manuscript.

**Keywords:** dental caries, oral disease prevention, oral health behaviour, national health surveillance, children

## Abstract

**Purpose:**

Improved oral health of children is noted in most Western countries, but this coincides with a high burden of oral disease in several countries of Central and Eastern Europe. The purpose of the present study was to describe the current level of dental caries in Hungarian children aged 5, 6, and 12 years and to assess the long-term trends in caries over 30 years. In addition, the report aims to highlight the oral health habits of 12-year-old children in Hungary.

**Materials and Methods:**

A representative survey was undertaken in 2016–2017 according to the WHO Pathfinder methodology, which was also applied in previous national oral health surveys of 1985, 1991, 1996, 2001, and 2008. Children of 5–6 and 12 years were sampled systematically in all surveys over 30 years. Data were gathered through clinical examinations and a questionnaire used for 12-year-olds.

**Results:**

In 2016–2017, 42.6% of 5- to 6-year-olds were caries free, with the proportion lower in rural than urban settings. Approximately four primary teeth were affected by caries among children aged 5–6 years. Most of the disease burden consisted of untreated caries. Caries experience was higher for children living in rural areas. At age 12, about two permanent teeth suffered from caries, and the D-component of the caries index was high. The percentage of caries-free 6-year-olds grew from 9% in 1985 to 42.6% in 2016-2017. In 1985, 12-year-olds had on average 5 teeth affected by caries, and after 30 years, the level of caries declined to 2.3 DMFT in 2016–2017. The responses to the questionnaire showed that 11.9% of 12-year-olds visited the dentist because of oral pain or discomfort and 40.5% were dissatisfied by the appearance of their teeth. About 40% of children consumed soft drinks or sweets/candy, several times a day.

**Conclusions:**

Hungary has not yet achieved the WHO goals for children aged 5–6. While Hungary accomplished the WHO goal for oral health of 12-year-olds by the year 2000, it is seems unrealistic for the country to achieve the WHO goal for 12-year-olds by the year 2020. For better oral health of children, strong emphasis should be given to population-directed oral disease prevention, including the reduction of sugar consumption and implementing public health programmes for the effective use of fluoride.

Evidence has accumulated in several Western European countries of declining prevalence and severity of oral disease in children.^5,7^ In particular, such a trend has been observed in the reduction of dental caries and improvement of gingival health conditions. The reasons for this progress are complex, but may be ascribed to better living conditions, strengthening of public health actions, and introduction of school oral health programmes focussing on disease prevention and health promotion.^2,8,12^ Certain countries have spent more resources on population-directed prevention for children in terms of systematic re-call intervals, effective administration of fluoride, promoting oral hygiene with the use of fluoride toothpaste, health education, and implementation of preventive services.^3,16,17^ Additionally, parents and caregivers tend to be very engaged in the oral health of children, which manifests in a more sensible approach to consumption of sugars and improved oral hygiene habits.^24^ Unfortunately, social determinants in children’s oral health persist and indicate significant inequities.^4^

The improved oral health conditions of the child population in most Western countries coincide with a high burden of oral disease in several countries of Central and Eastern Europe.^11,15,18,19^ The oral health care systems in this region are in transition after fundamental economic and political changes, and ongoing privatisation and decentralisation affects delivery of health services and oral health.^1^ Surveillance data are needed to assist the reorganisation of oral health care in Central and Eastern Europe. Except for Hungary, the epidemiological tradition is relatively weak in most of these countries and only a few national oral health surveys have been taken.

The World Health Organization (WHO) strongly recommends that regular national surveys be carried out to permit surveillance of oral health.^10^ In Hungary, the first standardised national survey of children was performed in 1985 and then followed by similar studies in 1991, 1996,^15^ 2001, and 2008.^13^ The most recent survey took place in 2016-2017. The purpose of the present project was to examine the current caries level in Hungarian children ages 5, 6, and 12 years and to assess the long-term trends in caries experience of the child population. In addition, the report aims to highlight the oral health habits of 12-year-old children.

## Materials and Methods

The survey in 2016-2017 was conducted according to the WHO Pathfinder methodology,^10^ which was also applied in the previous national oral health surveys.^15^ For all studies, identical examination areas were chosen to create a nationally representative sample. The areas were defined based on geographical location, urbanisation, expected caries levels, and fluoride content in drinking water. As part of the survey in 1985, samples of drinking water were collected from each of the examination sites. The water was analysed potentiometrically, showing that all fluoride values were fairly low (<0.25 mg F/liter).

In 2016–2017, children of 5 (n = 275), 6 (n = 377), and 12 years (n = 473) were sampled. As shown in Table 1, the 12-year-old children were covered in all samples since 1985. In 1985, children aged 6–7 years took part in the survey, while 5- and 6-year-old children were considered in 1991, 1996, 2001, 2008, and 2016-2017. Teams of calibrated dentists examined the children clinically according to the WHO criteria,^10^ using artificial light, dental mirrors, explorers, and the standard CPI periodontal probe. The clinical examinations of children involved the presence and severity of caries. Prior to the actual survey, examiners were calibrated in recording caries, yielding an inter-examiner consistency of 85% or more. Using the WHO Oral Health Questionnaire^10^ translated into Hungarian, the 12-year-olds were also asked about oral health-related behaviour. With guidance from the schoolteachers, the children themselves completed the self-administered questionnaire at school. Finally, following the dental examinations of children, interactive oral health education sessions were conducted in the schools and kindergartens, during which the children were instructed in proper toothbrushing and given other preventive advice.

**Table 1:** Overview of pathfinder surveys of 5–6- and 12-year-olds undertaken in Hungary by different years and numbers of children studied

Age	Year					
	1985	1991	1996	2001	2008	2016–2017
5–6 years	895 (6–7 years)	898	900	859	962	652
12 years	893	898	900	867	936	473

Clinical and questionnaire data were managed and analysed with SPSS Statistics Version 25 (SPSS; Chicago, IL, USA). Based on records from clinical examinations, dmft/DMFT indices were calculated according to WHO guidelines.^10^ Student’s t-test was used in the statistical evaluation of means, while the Chi-square test was used for comparison of proportions.

## Results

### Dental Caries

Table 2 shows the percentages of Hungarian children free of dental caries at ages 5-6 and 12 years. Four of ten children aged 5-6 years were caries free, with the proportion of children without caries being significantly lower in rural than in urban settings. Additionally, the present results reflected the presence of severe early childhood caries; the caries rate in the anterior primary dentition was 21.6%, with 14.6% in urban areas and 29.4% in rural areas.

**Table 2:** Percentage of caries-free Hungarian children aged 5–6 and 12 years by urbanisation

Age	Urban	Rural	Total
5-6 years***	49.9 (n = 343)	34.6 (n = 309)	42.6 (n = 652)
12 years	41.1 (n = 231)	38.0 (n = 242)	39.5 (n = 473)

***p<0.001

Among the 5- to 6-year-olds, approximately four primary teeth were affected by caries (Table 3). The vast majority of the disease burden consisted of untreated caries. The amount of caries was higher for children living in rural areas. At age 12, about two permanent teeth suffered from caries and the D-component of the caries index was prominent; however, the differences between rural and urban areas were minor. In addition, no variation in the occurrence of caries by urbanisation was found.

**Table 3:** Mean caries experience among Hungarian children aged 5-6 (dmft/DMFT) and 12 years (DMFT) in relation to urbanisation

	Urban	Rural	Total
5–6 years			
dt***	2.53 (3.56)	4.30 (4.31)	3.37 (4.03)
mt	0.06 (0.35)	0.07 (0.33)	0.06 (0.34)
ft***	0.23 (0.79)	0.05 (0.29)	0.14 (0.62)
dmft***	2.82 (3.73)	4.42 (4.37)	3.58 (4.12)
DMFT	0.10 (0.40) (n = 343)	0.10 (0.49) (n = 309)	0.10 (0.46) (n = 652)
12 years			
DT***	1.36 (1.84)	2.04 (2.55)	1.71 (2.26)
MT*	0.04 (0.31)	0.13 (0.44)	0.09 (0.38)
FT*	0.66 (1.35)	0.39 (1.20)	0.52 (1.28)
DMFT	2.07 (2.49) (n = 231)	2.55 (2.95) (n = 242)	2.32 (2.75) (n = 473)

* p < 0.05, ***p < 0.001 Standard deviation given in parentheses.

Table 4 indicates the percentages of children in need of specific intervention. Nearly one-third of 5- to 6-year-olds were in need of prompt curative care; notably, compared with urban children, twice the percentage of rural children had this treatment need. At age 12, one-third of children were found to need prompt curative care. For both age groups, a significant number of children required immediate treatment because of pain or infection.

**Table 4:** The percentages of Hungarian children aged 5-6 and 12 years in need for oral health intervention by urbanisation

	Urban	Rural	Total
5–6 years			
Prevention and routine curative treatment	13.7	10.4	12.1
Prompt curative treatment Immediate treatment due to pain or infection	19.8 4.7	40.8 *** 13.6 **	29.8 8.9
	(n = 343)	(n = 309)	(n = 652)
12 years			
Prevention and routine curative treatment	29.9 ***	16.5	23.0
Prompt curative treatment	32.9	37.2	35.1
Immediate treatment due to pain or infection	1.7	7.9	4.9
	(n = 231)	(n = 242)	(n = 473)

**p < 0.01, ***p < 0.001

### Trends in Dental Caries

**Fig 1:**
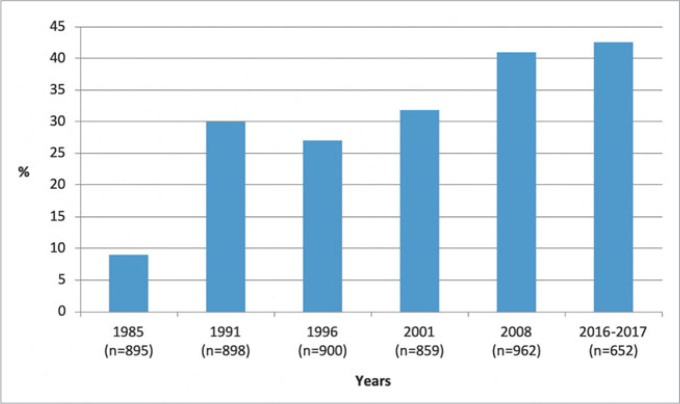
Percentage of caries-free 5- to 6-year-old children in Hungary, by year of survey.

**Fig 2:**
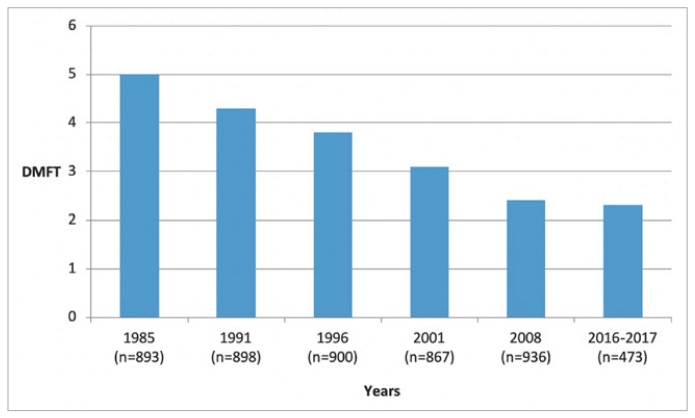
Mean dental caries experience (DMFT) of children in Hungary aged 12 years, by year of survey.

Figure 1 shows the changes over time in the percentage of caries-free 5- to 6-year-olds. In 1985, 9% of children were caries free, and the figure grew to 42.6% in 2016-2017. Figure 2 demonstrates the changes in caries experience of 12-year-old children. In 1985, children had an average of 5 teeth affected by caries, and the caries severity declined to a level of 2.3 DMFT in 2016-2017.

### Oral Health Behaviour

Table 5 presents the responses given by 12-year-olds to the questions about oral health-related behaviour. About half the children had seen a dentist several times during a year; 11.9% of the children reported that they were in pain or had troubles with their teeth, and four in ten children expressed dissatisfaction with the appearance of teeth. Half the children brushed their teeth twice a day or more. As to sugar consumption, the intake of sweets or candy, chewing gum with sugar, biscuits or cakes, soft drinks, and tea with sugar was frequent. Differences in oral health-related behaviour of children between urban and rural areas were not statistically significant.

**Table 5:** Percentage of Hungarian 12-year-old children (n = 473) with specific oral health related behaviour

Oral health behaviour	Item	Total
Dental visits during the past 12 months	Once	21.5
	2–3 times	34.6
	More than three times	20.6
Reason for last dental visit	Pain/trouble with teeth	11.9
	Routine check-up/treatment	55.1
	Treatment/follow-up treatment	17.4
Tooth cleaning	Once a day	44.5
	Two or more times a day	49.5
Perception of teeth or gums	Dissatisfied by appearance of teeth	40.5
	Avoid smiling and laughing because of my teeth	11.7
	Miss school class because of toothache or discomfort	9.8
Sugar consumption several times a day	Biscuits, cakes etc	31.5
	Sugary soft drinks	40.5
	Jam, honey	16.5
	Chewing gum with sugar	38.6
	Sweets, candy	42.3
	Milk with sugar	19.0
	Tea with sugar	37.1

## Discussion

In each year of study, the oral health survey of the two age groups was carried out according to the WHO Pathfinder methodology.^10^ As for the previous surveys, the findings of the current study may be considered representative for Hungary.^15^ In all surveys, exactly the same locations were included in the survey. However, the sample size in 2016-2017 was smaller. Nevertheless, the number of children selected for the two age groups was large enough to calculate precise statistical parameters. In conclusion, the design allowed a valid time-series analysis.

The present study demonstrated that about 40% of children were caries-free at the ages of 5 to 6; however, the proportion of caries-free children was markedly lower in rural than in urban areas. At age 12, caries experience was lower than that observed for other years of other studies.^13,15^ However, the volume of untreated caries remained relatively high, while the number of restored teeth was low. In parallel, a difference between rural and urban areas was noted with respect to caries experience of permanent teeth. Thus, the disease patterns confirm observations from the previous survey years, and they may reflect different living conditions in rural and urban areas. According to the clinical information, the need for intervention care was substantial for both ages, in particular among children living in rural areas, where 40% required prompt curative care. Moreover, a considerable number of these children required immediate treatment because of pain or infection.

Globally, the WHO has found that valid information on oral health-related behaviour may be obtained from children by the age of 12 years.^10^ Such data among children and parents could serve as an example for other countries with similar sociodemographic characteristics. The responses to the questionnaire revealed that the frequency of dental visits during the past 12 months was rather low, particularly in light of the considerable treatment need. Additionally, a large number of children reported being dissatisfied with their teeth, and some 10% stated that they missed school classes because of pain or discomfort. The recent WHO European Health of Youth Study^24^ established that 53.5% of Hungarian 13-year-olds brushed their teeth at least twice a day, and the present survey showed a similar toothbrushing frequency among 12-year-olds. Importantly, the present survey revealed that children consumed various sugary items several times a day, including sweets or candy as well as hidden sugar in assorted drinks. Such consumption does not comply with the decree established by the Ministry of Health in 2015 on healthy school cafeterias, which now involves a school directory concerning healthy foods and drinks. The reported high sugar intake is in agreement with the findings of the WHO study on the Health of Youth.^24^ In general, the current survey indicated a relatively high level of caries in rural children; however, only minor differences in oral health-related behaviour and consumption of sugars were observed between rural and urban children.

Gathering systematic health information over time is important for gaining knowledge about the processes and outcome of an oral health system, assessing the need for development or adjustment of programmes, and for sharing understanding and experience across countries. In European countries, surveillance indicators must be appropriate to the assessment of established oral health programmes, particularly to determine whether they are effective in reducing the burden of oral disease and promoting oral health. Since the 1980s, the WHO has encouraged countries to undertake health surveillance and formulate targets for children’s oral health. This has been promoted by the application of standard oral health indicators, which includes the two age groups of children surveyed in this study.^10^

The present series of surveys has created a surveillance scheme for the oral health of children in Hungary. The decline in caries prevalence has primarily been observed in Western countries, but the current report documented a similar long-term trend in Central Eastern European country. The improved oral health was shown over a significant span of 30 years and measured from the percentages of caries-free 5- to 6-year-olds, with a parallel 50% reduction in caries experience of 12-year-olds.

It is notable that the changing levels of caries coincide with a change in oral health programmes in Hungary. In 1985, the importance of oral and public health was emphasised by the Ministry of Health.^14^ A nationwide health programme, including oral health, was launched by an official decree, and national policies and goals were subsequently formulated in 1986. During the 1990s, the improvement of the national economy and health conditions promoted better knowledge and attitudes about prevention of oral disease among the general public, the availability of quality oral hygiene products, and the regular use of fluoride toothpaste increased considerably among children and families in Hungary.^13^ Furthermore, fluoride application was systematically provided by dental health professionals, better school environments were established, and healthier nutritional habits were encouraged along with better food served in schools. Numerous local community activities and preventive action programmes were organised, despite the oral health care system being in transition due to the dramatic economic and political changes involving privatisation and decentralisation. By 2003, a new national public health programme was introduced, with additional health promotion activities starting in 2010, 2015, and 2016. The new regulations towards healthy schools and adequate nutrition of children are now considered essential, particularly as the school has an important potential for breaking inequities in children’s health. However, it is unfortunate that community-based health programmes, including oral health through schools, have suffered from financial constraints in recent years.

### Challenges to Children’s Oral Health

The WHO European goals for oral health by the year 2000 state that in a given country, at least 50% of 6-year-old children should be caries free, and that 12-year-olds on average should have no more than 2 DMFT.^20^ The WHO European goals for the year 2020 state that in a given country, at least 80% of 6-year-old children should be caries free, whereas on average no more than 1.5 DMFT should be found in 12-year-old children.^21^ Hungary has not yet achieved the goals mentioned for 6-year-old children. While Hungary accomplished the goal for oral health of 12-year-olds by the year 2000, it seems challenging for the country to achieve the goal for 12-year-olds by the year 2020. Therefore, strong emphasis should be given to application of well-known strategies in population-directed oral disease prevention, particularly through the reduction of sugar consumption.^6,22,23^ In a general perspective, interdisciplinary work in disease prevention is a vital approach to promoting the health of all children in Hungary. The efforts towards increased use of toothpaste containing fluoride has been successful^14^ and it is anticipated that implementing automatic fluoridation programmes^9,25^ may be important for further caries reduction among children in Hungary.
